# Standardization of a High‐Quality Methodological Framework for Long‐Term Genetic Monitoring of the French Wolf Population

**DOI:** 10.1002/ece3.71345

**Published:** 2025-04-24

**Authors:** Agathe Pirog, Christophe Duchamp, Cécile Kaerle, Caroline Dufaure de Citres, Sabine Rousselot, Juliette Lavarec, Guillaume Queney

**Affiliations:** ^1^ ANTAGENE, Animal Genomics Laboratory La Tour de Salvagny (Lyon) France; ^2^ Department of Research and Expertise French Agency for Biodiversity Nantes France; ^3^ Department of Research and Expertise French Agency for Biodiversity Gap France

**Keywords:** *Canis lupus*, France, gray wolf, long‐term monitoring, microsatellite, mitochondrial control region, noninvasive samples

## Abstract

Since the gray wolf was eradicated from large parts of Europe, this species has been recolonizing much of its former distribution, particularly since the past 30 years. Wolves benefit from European legal protection through the Habitats Directive and the Bern Convention, and reporting on the evolution of their populations in each country of Europe is mandatory. To monitor French wolf populations over the long term, a standardized high‐quality methodological framework has been developed to analyze data from noninvasively collected samples and assess population diversity. We delineated each step and implemented a laboratory control procedure to analyze 8733 samples harvested within the French distribution range of the species between 2006 and 2022, and provided key quality and diversity indicators. Of these samples, 82.8% were successfully amplified and sequenced for the mitochondrial control region. Subsequently, the wolf samples were genotyped at 22 microsatellite autosomal loci and a sex locus displayed over two independent multiplexes using the multitube approach. The average success rate of polymerase chain reaction per locus was 64.2% across all replicates. The residual genotyping error rates were low compared to those in other studies using non‐invasively collected samples, with mean residual allelic dropout rates of 5.8% per locus and mean residual false allele rates of 1.0% per locus. The high‐quality dataset identified 1735 individuals in total over the last 15 years, of which 99.9% exhibited a single Italo–Alpine mitotype. Genetic diversity was relatively low, with mean observed heterozygosity of 0.482 and mean expected heterozygosity of 0.519. This supports the natural colonization of the French Alps by a few individuals originating from the remaining Italian populations, which started approximately 30 years ago. By generating high‐quality standards and quality control processes, this protocol enhances the cost‐efficiency ratio of monitoring French wolf populations and holds high value for managers tasked with the management and conservation of wolf populations in the long term.

## Graphical abstract

This study aimed to describe the essential steps and control procedures for establishing a high‐quality, standardized methodological framework for long‐term monitoring of the French wolf population. We described a panel of 22 microsatellite loci, together with a sex marker, and estimates of key quality indicators for evaluating the framework. Finally, we used this panel to estimate the basic indicators of genetic diversity in the French wolf population.

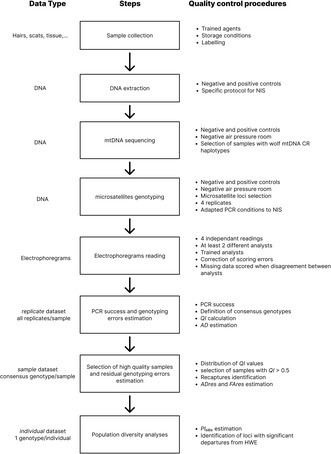

## 1 Introduction

The gray wolf, *Canis lupus* , was widespread in Europe. After becoming almost extinct in the western part of the continent, the conservation measures implemented in the second half of the 20th century, combined with an increase in ungulate populations that this species preys on, have favored gradual recolonization of several wolf populations in Western Europe (Chapron et al. 2014). Because the species is legally protected by the Habitats Directive and Bern Convention, each European country needs to report the evolution of gray wolf populations in its territory, which requires intensive and long‐term monitoring. In the French Alps, the first individuals were observed in the early 1990s, after migrating from the Italo–Alpine population (Valière et al. 2003; Fabbri et al. 2007). Since then, the population has been closely monitored by the French authorities for assessing the evolution of its size and spatial distribution over time, and the occurrence of depredation on livestock (Cubaynes et al. 2010; Louvrier et al. 2018; Grente et al. 2022). Legal kills derogations are conducted by the authorities under specific conditions following the national action recommendations, primarily to protect livestock from predation (MTES MAPAAR 2019; https://www.auvergne‐rhone‐alpes.developpement‐durable.gouv.fr/IMG/pdf/nap_wolf_and_stock‐rearing_activities_2018‐2023.pdf).

To this end, extensive sampling has been carried out every year for more than 30 years to collect noninvasive samples (Duchamp et al. 2012). Noninvasive samples, such as hair, feces, urine, and saliva, are increasingly recognized as important sources of information for monitoring wildlife conservation. Extracting DNA from these samples has provided insights into the population dynamics and ecology of elusive species, such as gray wolves, while minimizing disturbances to their behavior and habitat (Mills et al. 2000; Waits and Paetkau 2005; Schwartz et al. 2007). In particular, noninvasive sampling has been extensively used to gain insights into the phylogeographic history of gray wolf populations (Valière et al. 2003), estimate population sizes (Creel et al. 2003; Petit and Valière 2006; Cubaynes et al. 2010; Caniglia et al. 2012), investigate hybridization with dogs (Sundqvist et al. 2008; Godinho et al. 2015), and monitor reintroduced populations (Stenglein, Waits, et al. 2010). Therefore, noninvasive samples collected in France over the last 30 years can be used to answer these questions regarding the French gray wolf population. In particular, they have been used to estimate annual population size using recapture methods (Cubaynes et al. 2010; Louvrier et al. 2018). However, the use of such samples requires standardized protocols for performing specific analyses and maintaining a consistent workflow over time while controlling for genotyping errors.

DNA obtained from noninvasive samples is often degraded or present in very low quantities, increasing the risk of DNA contamination, amplification, and genotyping errors (Taberlet et al. 1999; Broquet and Petit 2004; Waits and Paetkau 2005), which adversely affect the estimation of population dynamics. Genotyping errors are well known and can be divided into two types: amplification failure of one of the two alleles of an individual (allele dropout; AD) and polymerase errors, amplification artifacts, or contamination leading to an additional allele (false allele; FA), with the latter occurring less frequently than the former (Gagneux et al. 1997). To overcome these challenges, several methods and protocols have been proposed, which highlight crucial steps in data processing from noninvasive samples (Frantz et al. 2003; Bonin et al. 2004; Beja‐Pereira et al. 2009; Lampa et al. 2013). These steps include (i) selecting the molecular markers to be used, (ii) limiting the occurrence of genotyping errors, and (iii) evaluating the residual error rates and data quality.

Microsatellite loci are still widely used when monitoring species using noninvasive samples, because of their high polymorphism (Selkoe and Toonen 2006), and the transition from microsatellite loci to single nucleotide polymorphism poses a challenge for long‐term monitoring programs (Osborne et al. 2023). However, the use of microsatellite loci requires the scoring of alleles, whether manually or by automated and semi‐automated scoring of fluorescence profiles, a process that is particularly error‐prone (Paetkau 2003; Pompanon et al. 2005). Therefore, several studies have recommended that alleles be scored independently by multiple trained analysts (Beja‐Pereira et al. 2009, Lampa et al. 2013).

Genotyping errors can be reduced by performing independent polymerase chain reactions (PCRs) with multiple replicates per sample, which are known as the multitube approach (Taberlet et al. 1996).

In addition, various statistical analyses can be performed on the collected dataset for assessing genotyping errors and evaluating data quality. First, the AD and FA rates can be estimated by comparing replicated genotypes with the consensus genotype (Valière 2002; Broquet and Petit 2004). Similarly, the residual rates of AD and FA can be estimated by comparing genotypes from different samples of the same individual. Methods using reconstructed pedigrees (Wang 2018) and estimates of homozygous excess at one or more molecular markers (Van Oosterhout et al. 2004; Dąbrowski et al. 2015) have also been developed. Sample quality can also be assessed by calculating the standardized quality indices based on estimates of amplification success and genotyping errors (Miquel et al. 2006). These analyses facilitate the quantification of residual error rates in the dataset, identification of error‐prone markers or samples, and evaluation of the performance of the applied methodology.

While all these control procedures have already been documented in studies using noninvasive samples, specifically in studies focusing on gray wolf populations (Creel et al. 2003; Scandura et al. 2006; Stenglein, Waits, et al. 2010; Caniglia et al. 2012; Nakamura et al. 2017; Štikarová et al. 2019), they have rarely been described in a single and continuous workflow. In addition, the control procedures described in the literature needed to be adapted to extensive noninvasive sampling in France (> 11,000 noninvasive samples collected between 1992 and 2024) to provide annual estimates of population size in short time periods in a cost‐effective monitoring framework.

This study aimed to describe the essential steps and control procedures for establishing a high‐quality, standardized methodological framework for long‐term monitoring of wolves and other elusive species, using noninvasive samples. We applied this framework to the French wolf population as an example species. We described a panel of 22 microsatellite loci, together with a sex marker, and estimates of key quality indicators for evaluating the framework. Finally, we used this panel to estimate the basic indicators of genetic diversity in the French wolf population.

## 2 Study Area

The study area covers the whole France but extends mainly from the Pyrenees in the southeastern part to the Vosges in the northern upper part of France (102,483 km^2^). The gray wolves cover various ecological and landscape features, including mountainous regions in the Alps and lowland areas, since the last phase of gray wolf recovery (Duchamp et al. 2012). The ungulates preyed upon by the gray wolf are mainly roe deer ( *Capreolus capreolus* ), red deer ( *Cervus elaphus* ), and wild boar ( *Sus scrofa* ) in lowlands, and chamois ( *Rupicapra rupicapra* ), mouflon ( *Ovis musimon* ), and deer in the mountain regions. Population density in the study area varies between 30 and 200 inhabitants per km^2^ depending on the department. Approximately 1,500,000 sheep are bred annually in the area for meat and milk production (IDELE 2018). During summer, a large proportion of these herds become transhumant and move to high‐altitude pastures in the Alps. Gray wolf attacks on livestock are documented every year, with some regular hotspots (Gervasi et al. 2021; Grente et al. 2022), particularly in the Alpine region, leading to significant economic loss (Linnell and Cretois 2018).

## 3 Methods

### 3.1 Sampling

Since 1994, gray wolf monitoring has been carried out throughout the year in the presumed range of the gray wolves in France using sign presence surveys. A survey at two spatial scales with different sampling strategies is sequentially conducted each year (Duchamp et al. 2012). The French Agency for Biodiversity (OFB) oversees the sampling and is supported by trained volunteers, including OFB representatives, gamekeepers, natural park managers, trained hunters, naturalists, sheep farmers, and mountain professionals. This network of people aims to conduct (i) a large‐scale signal survey to detect the occurrence of new gray wolves, and (ii) an intensive signal survey within a known gray wolf territory to monitor group size and composition (Duchamp et al. 2012). All data are then used to produce annual updates of the demographic pattern and to estimate population size (Cubaynes et al. 2010; Marescot et al. 2011). All observers are trained in collecting noninvasive samples such as feces, urine, or hair. Those responsible within the core area (the known gray wolf area) are asked to select the most recent samples and discard feces samples that appear to be older than 2 weeks. Observers outside the core area are expected to collect all suspected samples unfiltered to maximize the detection of dispersal events. Observers collect scat and hair primarily along trails or rural roads, along transects or opportunistically. Urine samples are collected along the tracks left by gray wolves in snow‐covered areas. In addition, tissue or blood samples are collected from individuals killed during control operations by French government authorities (fresh samples) and carcasses of gray wolves that died from traffic collision, illegal killing, or other causes. Fecal and tissue samples are stored in hermetic ZIPloc bags at −20°C as soon as they are brought from the field, before being stored under laboratory conditions in 20‐mL 96% ethanol pillboxes. Individual gloves and sterilized tools are used for each sample to avoid contamination. Urine and blood samples are conditioned as described by Valiere and Taberlet (2000), and hair samples are stored in an envelope with dry air. All samples are stored in a single national database with a unique reference number to ensure traceability.

**FIGURE 1:**
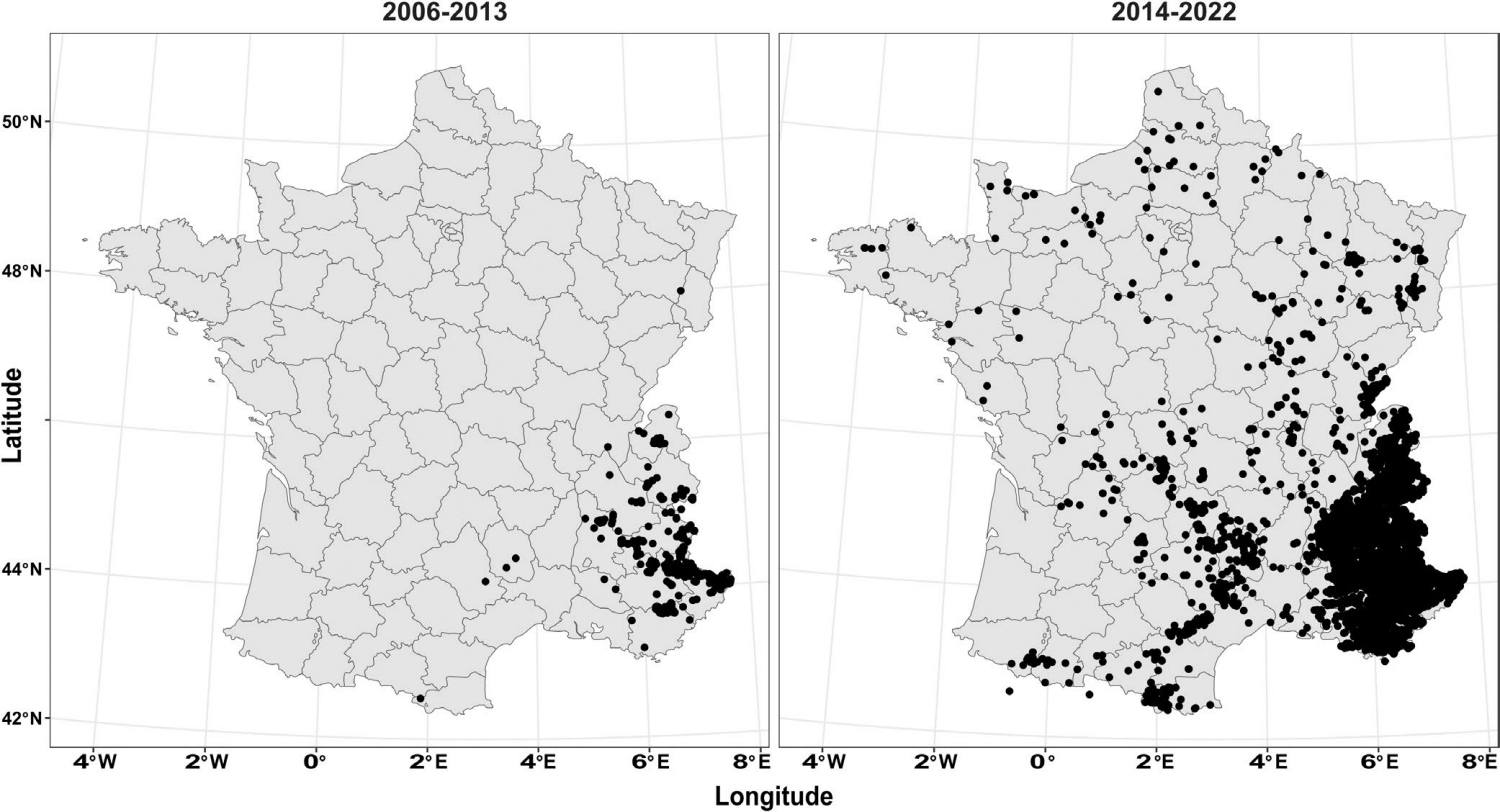
Evolution of the sampling coverage performed to monitor the French gray wolf population from 2006 to 2022 (*n* = 8733 samples).

In this study, 8733 samples were analyzed in 45 independent laboratory sessions, including 7316 fecal samples (84%), 609 tissue samples from dead wolves, 543 urine samples, 177 hair samples, and 88 blood samples collected between 2006 and 2022 (Figure 1). Tissue and blood samples were included for quality comparison with non‐invasive samples and to provide a comprehensive representation of the French gray wolf population. Each year, all samples from the colonization area are primarily selected to detect new potential dispersal events or packs. Subsequently, samples from the core area are selected to cover the annual monitoring of each identified pack or mating territory.

### 3.2 Workflow

**FIGURE 2:**
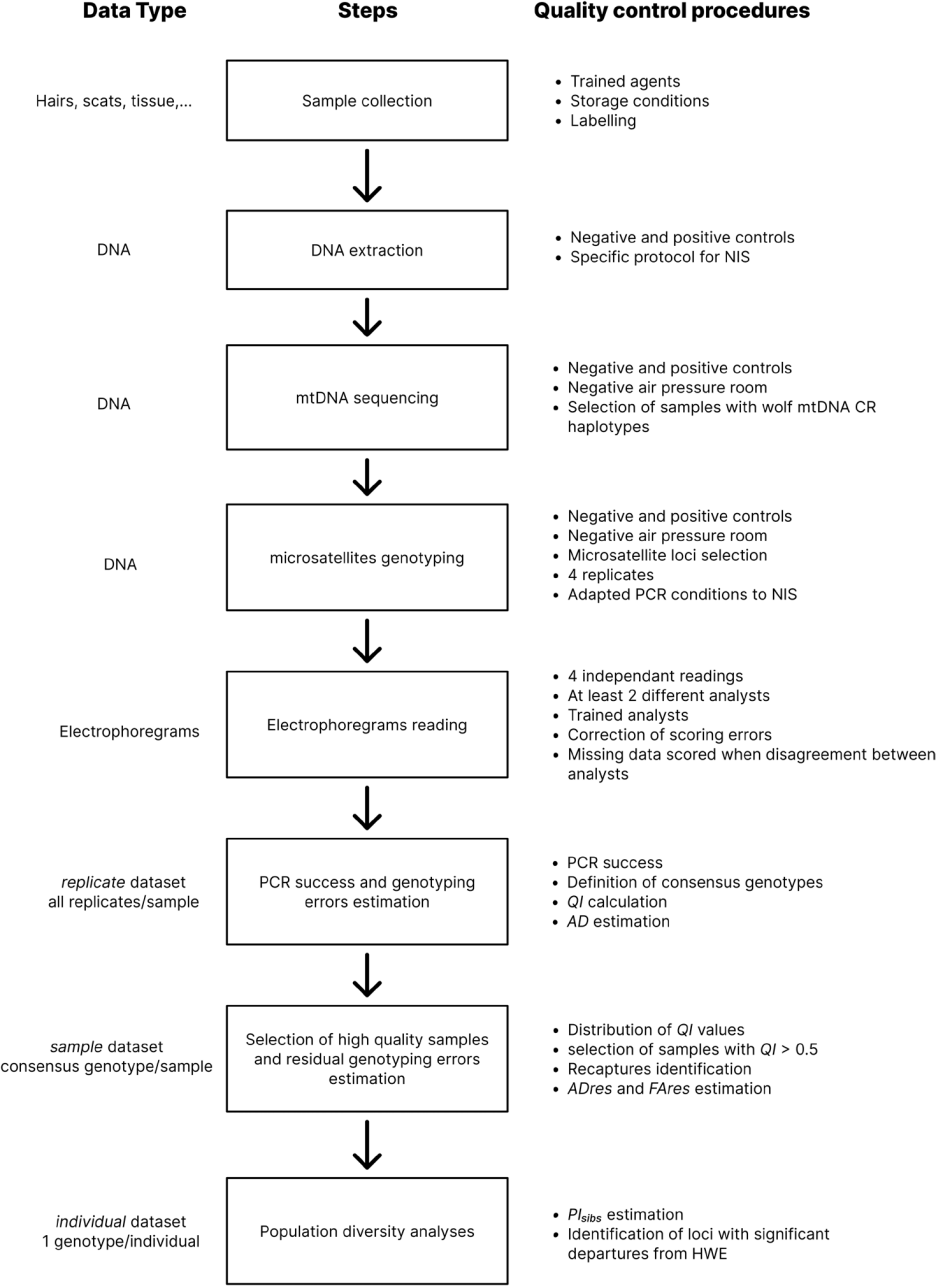
Flowchart of critical steps and control procedures applied to process gray wolf noninvasive samples. AD, Allelic dropout rate; AD_res_, Residual allelic dropout rate; FA_res_, Residual false allele rate; HWE, Hardy–Weinberg Equilibrium; MtDNA CR, Mitochondrial Control Region; NIS, Noninvasive samples; PI_sibs_, Probability of identity among siblings; QI, Quality Index.

A unidirectional workflow was followed for each sample (Figure 2) with dedicated spaces for rare and degraded DNA in each step. First, the samples are processed upon receipt to verify the consistency of information with the corresponding file. They are then prepared for DNA extraction, which takes place in a special room for noninvasive samples with an ultraviolet light purification system. Extracted DNA samples are stored in freezers at −20°C. Sensitive reagents (enzymes and DNA primers, etc.) used for DNA amplification are prepared in a clean room with positive air pressure, accessible via entrance and exit airlocks. DNA and reagents are then assembled (manual and automated work; pre‐PCR step) before amplification and analysis of amplified DNA (post‐PCR step). The post‐PCR step is performed in a large clean room with negative air pressure (accessible via two input and output airlocks), which allows continuous air recycling (independent air supplier) and is equipped with thermocycler machines and ABI‐3730xl DNA Analyzers (Applied Biosystems, Foster City, CA, USA).

### 3.3 DNA Extraction

Sterile disposable tools were used to manipulate samples, and the workstation was cleaned with bleach after processing each sample to avoid cross‐contamination. Each tissue sample was transferred to a sterile and labeled microtube for DNA extraction. For feces, urine, and blood, the supernatant was first transferred to a numbered microtube, centrifuged to remove ethanol, and then DNA extraction was performed from the pellet. DNA from tissue and noninvasive samples was separately extracted. Two negative extraction controls (blanks) and one positive extraction control (sample, feces or tissue, depending on the type of sample extracted, previously analyzed and validated for DNA quality and genotyping success with microsatellite markers) were added to each 96‐well plate. Samples were lysed together with the positive and negative extraction controls overnight at 56°C according to the manufacturer's instructions (Nucleospin 96 Tissue Kit; Macherey‐Nagel, Düren, Germany). DNA was isolated and purified using purification columns and vacuum filtration (Nucleospin 96 Tissue Kit). DNA samples were stored in labeled 96‐tube strip plates at −20°C in a freezer.

### 3.4 Mitochondrial Sequencing and Lineage Identification

To identify the species and mitochondrial haplotype of gray wolf samples, 605 bp of the mitochondrial control region (mtDNA CR) was amplified by PCR using the primers CR‐Thr‐L‐15926‐F and CR‐DL‐H‐16340‐R (Vilà et al. 1999).

PCR amplifications were performed in 96‐well microplates with a 10 μL final volume containing 5 μL Mastermix Taq polymerase (Type‐It PCR Kit; Qiagen, Hilden, Germany), 0.20 μM nonfluorescent CR primer pair, and 3 μL DNA extract. The thermal cycle was as follows: 95°C for 5 min, followed by 40 cycles at 95°C for 30 s, 52°C for 90 s, and 72°C for 45 s, followed by extension at 60°C for 10 min. Three control PCR blanks and three positive control DNA samples were added to each 96‐well microplate.

The PCR products were bidirectionally sequenced by the Sanger method using a BigDye Terminator v.3.1 Cycle Sequencing Kit (Applied Biosystems, Foster City, CA, USA) with the same primers. After purification, the sequences were analyzed using an ABI PRISM 3130 XL capillary sequencer (Applied Biosystems, Foster City, CA, USA), and the electropherograms were interpreted using SeqMan Pro (DNASTAR, Madison, WI, USA).

For each sample, the resulting consensus sequence was compared to both public databases and wolf lineage haplotypes (Pilot et al. 2010) using BLAST (http://blast.ncbi.nlm.nih.gov/Blast.cgi). The identity scores and E‐values were then analyzed to determine which species each sample belonged to and which haplotypes the gray wolf samples exhibited. Only samples with haplotypes specific to gray wolves were retained. Samples with haplotypes identified by Pilot et al. (2010) and known to be shared by domestic dog, *Canis familiaris* , and gray wolf, *C. lupus*, were identified as *Canis* species. Since we could not confidently confirm that these samples originated from wolves and they may have come from wandering dogs, we chose not to include them in further analyses.

### 3.5 Microsatellite Genotyping

DNA was genotyped using 22 autosomal microsatellites and a marker for sex identification (Amelogenin, heterozygote for males and homozygote for females; Ji et al. 2007) distributed in two multiplexes (Appendix 1). The set of 22 microsatellites included 11 loci selected because they can detect hybridization events between domestic dog and gray wolf (Godinho et al. 2015), while the remaining loci are commonly used in genetic studies of gray wolf populations in Europe (Lucchini et al. 2002, 2004; Pilot et al. 2006; Fabbri et al. 2007; Sastre et al. 2011; Jansson et al. 2012; Hindrikson et al. 2013).

PCR reaction mixtures were prepared according to the unidirectional workflow described above. After preparing the sensitive reagents (enzymes and DNA primers), DNA and reagents were mixed using filtered tips in a pre‐PCR room. Three control PCR blanks and three positive control DNA samples were added to each 96‐well microplate. PCR amplifications were performed in the designated post‐PCR room in a final volume of 10 μL containing 5 μL master mix Taq Polymerase (Type‐It Microsatellite PCR kit; Qiagen, Hilden, Germany), 0.63 or 0.66 μL of one of the two pools of 12 primer pairs (for each of the two multiplexes) at a concentration of 0.05–0.8 μM depending on the multiplex, and 3 μL DNA. One primer of each pair was coupled to a fluorescent dye (Appendix 1).

The thermal cycle was as follows: 95°C for 5 min, followed by 40 cycles at 95°C for 30 s, 58°C for 90 s, and 72°C for 45 s, and extension at 60°C for 30 min. For fresh tissue samples, the number of cycles was reduced to 35. The PCR products were resolved using an ABI PRISM 3130 XL capillary sequencer (Applied Biosystems) under denaturing conditions (formamide) with an internal size marker in one migration for each multiplex. The electropherograms for each sample were analyzed using GENEMAPPER v.4.1 (Applied Biosystems).

Four replicates were systematically performed for each noninvasive sample and two for tissue samples of recently killed wolves. Indeed, simulations showed that increasing the number of replicates beyond four for noninvasive samples improved genotype accuracy only for medium‐quality samples. Low‐quality samples remained low quality regardless of the number of replicates, while four replicates were sufficient for good‐quality samples (data not shown). At least two different analysts independently checked the electropherograms of each replicate. In the event of a discrepancy between the analysts at one locus, the results were contested. If, after discussion, no consensus could be reached, a missing data for the locus in question was scored. These analysts were trained to accurately assign alleles to the selected loci based on previously analyzed tissue samples validated for DNA quality and genotyping success, and samples with degraded DNA. They were able to distinguish true alleles from artifacts removed during scoring. If two true alleles were observed at a locus in one of the replicates or across all replicates of a sample, the sample was considered contaminated and was not used for further analyses. Full agreement between the analysts across the entire set of loci was required to validate genotyping scoring.

A consensus genotype for each sample was created based on the genotypes observed in each replicate. We considered each amplified allele that was conserved by the analysts as a true allele and retained it in the consensus genotype (FA was corrected during scoring), that is, one amplification for each allele required for heterozygous and homozygous loci.

### 3.6 Quality Analyses

Statistical analyses were performed on the three datasets (Figure 2) to estimate quality indicators and population diversity statistics.

- *Sample replicate dataset*: One row per replicate per sample.
- *Sample‐consensus dataset*: One row per consensus genotype of 2–4 replicates.
- *Individual consensus dataset*: One row per individual.

#### 3.6.1 PCR Success, Sample Quality, and Individual Identification

First, the *Sample replicate dataset* was used to evaluate the success rate of PCR and sample quality. PCR success per locus was calculated as the number of successful amplifications divided by the total number of attempted amplifications.

Then, sample quality was assessed by calculating a quality index (QI) for each locus and comparing the genotype observed in each replicate with the consensus genotype (Miquel et al. 2006). A score of 1 was assigned to the replicate if its genotype for the locus under consideration matched the consensus genotype; 0.5 if an AD was present; and 0 if the genotype was missing (i.e., no amplification, contamination, or disagreement between analysts). The mean of these scores was calculated for the locus across all replicates. Then, the mean of these scores was calculated across all loci for each sample, resulting in a QI ranging from 0 (poor quality) to 1 (excellent quality).

Then, the dataset containing only the consensus genotypes for each sample (*Sample consensus dataset*) was used for examining the distribution of the mean QI scores per sample. High‐quality samples, that is, with QI values > 0.5 (threshold chosen after preliminary analyses; data not shown), were used to identify different individuals. Samples with identical consensus genotypes (missing data at a locus were considered similarity) and sharing at least 12 loci with no missing data were assigned to a single individual. Furthermore, samples from the same individual may exhibit allele mismatches due to AD or FA. The probability of observing a mismatch follows a binomial distribution B(*n, s, p*), where *n* is the number of loci, *s* is the number of mismatches, and *p* is the mean allelic dropout rate for high‐quality samples. The probability of observing at least one error across 12 loci was 0.587, with 99% of errors falling in the range of one to three mismatches. Based on these analyses, samples with three or fewer allele mismatches were assigned to the same individual. Additional information such as sample quality, date, and location of sample collection was used to confirm whether the two samples should be assigned to the same individual. We retained a consensus genotype for each individual for defining individual multi‐locus genotypes.

Finally, the *Individual consensus dataset* was used to estimate the performance of the microsatellite loci set in identifying distinct individuals. We calculated the probability that two individuals presented the same multi‐locus genotype by chance, that is, the probability of identity between siblings (PI_sibs_) (Waits et al. 2001), for each combination of loci, which ranged between 2 and 22 loci.

#### 3.6.2 Estimation of Genotyping Errors

The *Sample replicate dataset* was used to estimate the initial allelic dropout rate for each sample and locus, as described in equation 1 by Broquet and Petit (2004), that is, by dividing the number of observed AD by the number of successful PCRs, considering only replicates from samples with heterozygous consensus genotypes. We estimated only the AD rates at this stage, as analysts corrected for FA when reading the electropherograms. This was performed with replicates of all samples (AD) and after selecting replicates of high‐quality samples only (AD_high_). The *Sample consensus dataset* and consensus genotypes of the recaptured individuals were then used to estimate the residual AD (AD_res_) and FA (FA_res_) rates. The genotypes from different samples assigned to the same individual were compared with the consensus genotype retained for the individual. For AD_res_, equation 1 by Broquet and Petit (2004) was used, similar to the *Sample replicate dataset*, while FA_res_ was estimated by dividing the number of observed FA by the number of successful amplifications for each locus (equation 3 by Broquet and Petit 2004). These indicators quantified the remaining allele dropouts and false alleles in the dataset after correcting for those detected in the replicates of each sample.

Finally, the remaining AD in the *Individual consensus dataset* was assessed using Micro‐Checker (Van Oosterhout et al. 2004) and ML_NullFreq (Kalinowski and Taper 2006).

### 3.7 Population Genetic Diversity

Using the *Individual consensus dataset*, the observed and expected heterozygosity (*H*_*o*_ and *H*_*e*_), fixation index (*F*_IS_), and number of alleles per locus were assessed using the R package hierfstat (Goudet and Jombart 2015). Significant departures from Hardy–Weinberg equilibrium (HWE) were calculated by permuting alleles within the population using 1000 permutations (Goudet and Jombart 2015).

Statistical analyses (except for those performed with Micro‐Checker and ML_NullFreq) were performed using R (R Core Team 2023) and RStudio (Posit Team 2024).

## 4 Results

### 4.1 Mitochondrial Sequencing and Lineage Identification

**FIGURE 3:**
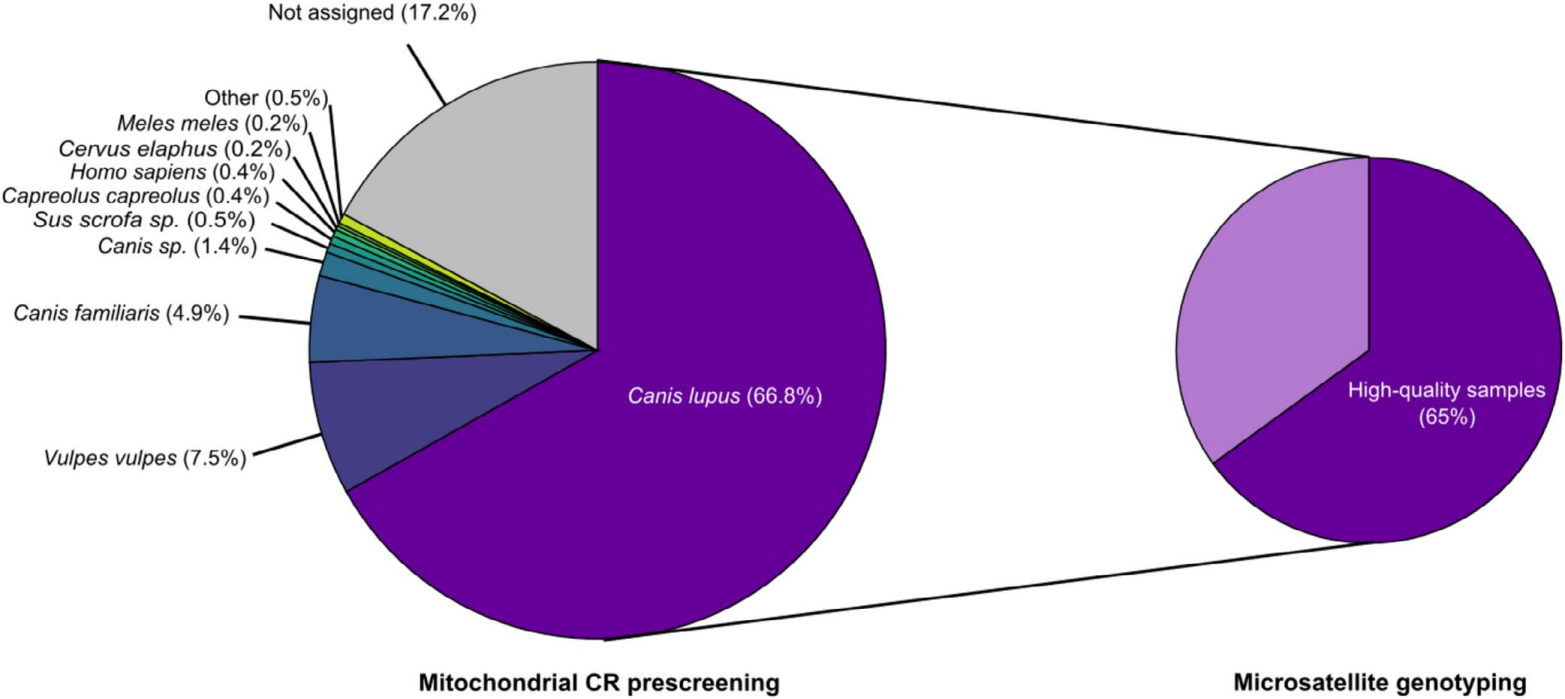
Proportion of samples collected for monitoring the French gray wolf population that were successfully analyzed using the mitochondrial control region and the panel of 22 microsatellite loci. CR, Control region. The size of the circles is proportional to the number of samples collected (left circle, *n* = 8733 samples) and the number of samples with a gray wolf haplotype (right circle, *n* = 5840). The left circle shows the percentage of samples for which a species could be identified when sequenced at the mitochondrial control region, and the right circle shows the percentage of gray wolf samples that had a Quality Index (QI) > 0.5 and were not contaminated when genotyped with the panel of 22 microsatellite loci.

Complete mtDNA CR sequences were obtained for 7234 samples (82.8%; Figure 3) out of the 8733 samples analyzed (1499 samples with no species assigned). Of these 7234 samples, 5840 were assigned without ambiguity to *C. lupus* (66.8% of the samples collected; Figure 3). Of the 117 samples assigned to *Canis* sp. (1.4% of samples collected), seven had mitotypes shared by domestic dog and gray wolf (one w2, four w7, and two w14 mitotypes, as defined by Pilot et al. [2010]), whereas the mitochondrial CR sequence in 110 other samples was not of sufficient quality for identifying the species.

Among the 5840 gray wolf samples, the Italo–Alpine lineage was identified in 5832 samples (99.9%), presenting the w22 mitotype identified by Pilot et al. (2010). Six other samples were assigned to the eastern European lineage, presenting the w1 mitotype, as identified by Pilot et al. (2010). The two remaining samples presented the same mitotype, which corresponded to a sequence referenced in GenBank (accession number KF661072) and was obtained from a sample collected in Alaska. These samples corresponded to an individual that escaped from captivity after a storm and was detected in the wild in the two following years and never since.

### 4.2 Quality Analyses

#### 4.2.1 PCR Success, Sample Quality, and Individual Identification

**FIGURE 4:**
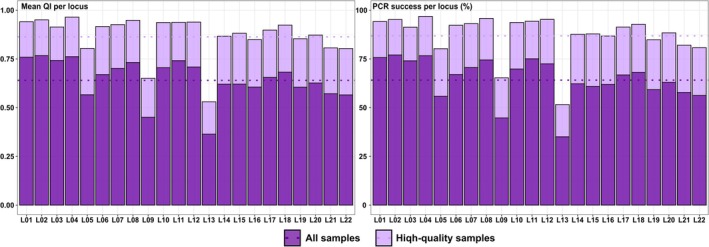
Quality Index values and PCR success per locus for the gray wolf samples collected in France. For each indicator, darker purple bars indicate values estimated using all genotyped samples (*n* = 5840), while lighter purple bars denote values estimated using only samples with Quality Index (QI) values > 0.5 and without contamination (*n* = 3797). The mean of the indicator across all loci is represented by the dotted line.

The *Sample replicate dataset* included replicates of 5840 genotyped samples. Using this dataset, the mean PCR success rate per locus was 64.1% (Figure 4; Appendix 2), and the mean QI value per locus was 0.64 (Figure 4; Appendix 2).

**FIGURE 5:**
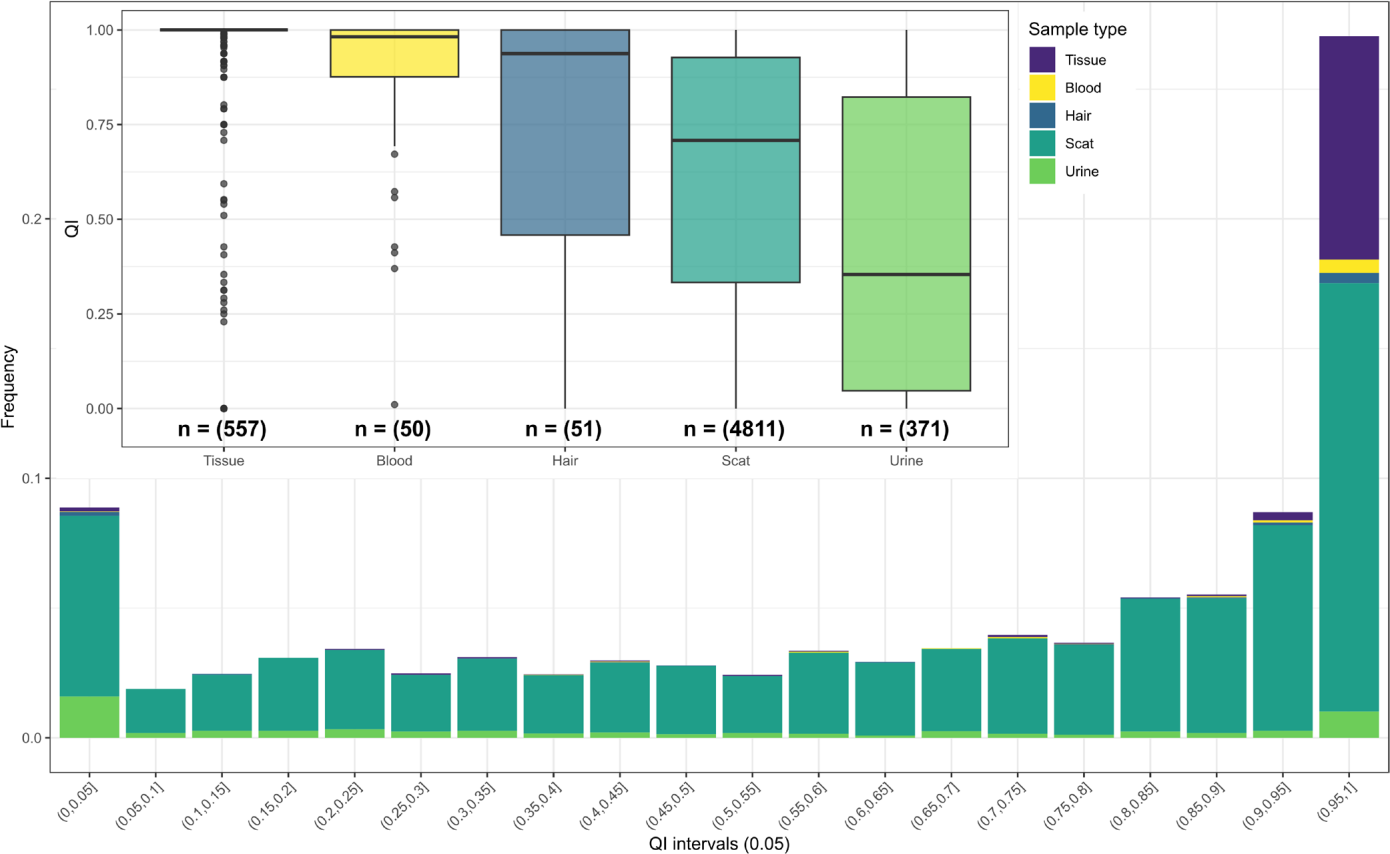
Quality indices distribution among gray wolf samples collected in France (*n* = 5840). Boxplots indicate mean Quality Index (QI) values per sample type and colors correspond to sample type (tissue, blood, hair, scat, or urine).

Using the *Sample consensus dataset*, amplifications were not observed in 473 samples (8.1% of the genotyped samples), primarily consisting of scats (Figure 5). Furthermore, 122 samples presented possible contamination (2.1% of the genotyped samples). The mean QI values per sample varied between 0 and 1, with 50% of the samples presenting QI values above 0.76 and 25% above 0.96 (Figure 5). Tissue and blood samples generally demonstrated better quality than the other sample types, with mean QI values of 0.96 and 0.88, respectively (Figure 5), although some tissue samples were degraded, indicated by mean QI values < 0.75 (27 samples; 4.8% of tissue samples). These samples mainly corresponded to those collected from wolves found dead and already degraded (only four fresh tissue samples among them).

We retained samples with mean QI values > 0.5 and without evidence of contamination for performing subsequent analyses, as we found this QI threshold to be a good compromise between enhancing the quality of the retained samples without discarding too many samples (previous analyses showing less than four missing loci per genotype on average). These samples (*n =* 3797; 65.0% of the genotyped samples) presented a mean PCR success rate per locus of 87.3% and a mean QI value per locus of 0.87 (Appendix 2). They were used to determine the number of distinct individuals.

Out of these 3797 samples, the three mismatches threshold identified 1735 distinct individuals sampled over 15 years, of which 1054 were sampled only once, and 681 were sampled 2–35 times, with a mean of 4.03 samples per individual.

**FIGURE 6:**
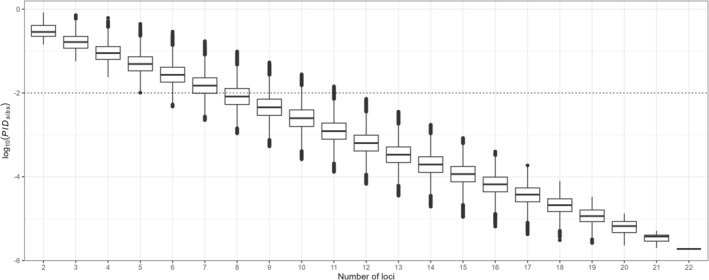
Probability of Identity among siblings (PI_sibs_) values calculated using the 1735 different individuals identified. The dotted line delineates the 1.00 × 10^−2^ threshold.

Using the *Individual consensus dataset* (1735 distinct individuals), the discriminatory power of the 22 microsatellite loci for identifying distinct individuals was high, with a PI_sibs_ of 1.87 × 10^−6^ (Figure 6). After testing all possible combinations of loci, successful genotyping of at least nine loci was necessary to ensure that the mean PI_sibs_ value was below the 10^−2^ threshold. However, to consistently remain below this threshold regardless of the included loci, successful genotyping of at least 12 loci achieved a PI_sibs_ of maximum of 7.22 × 10^−3^ for the least variable combination (Figure 6 and Appendix 3). Furthermore, the 3797 samples used for individual identification yielded around 7.2 million comparisons, of which 6756 (0.09%) concerned genotypes with 12 shared loci without missing data. With a mean PI_sibs_ of 6.35 × 10^−4^ for 12 successfully genotyped loci, the threshold of 12 loci with no missing data used to assign samples to a single individual was thus adapted. In addition, when requiring at least 16 shared loci without missing data between samples assigned to the same individual (i.e., mean PI_sibs_ of 6.52 × 10^−5^), the estimated number of individuals remained nearly unchanged with a decrease of less than 0.9%. This indicates a low level of uncertainty in the identification of samples originating from the same individual.

#### 4.2.2 Estimation of Genotyping Errors

**FIGURE 7:**
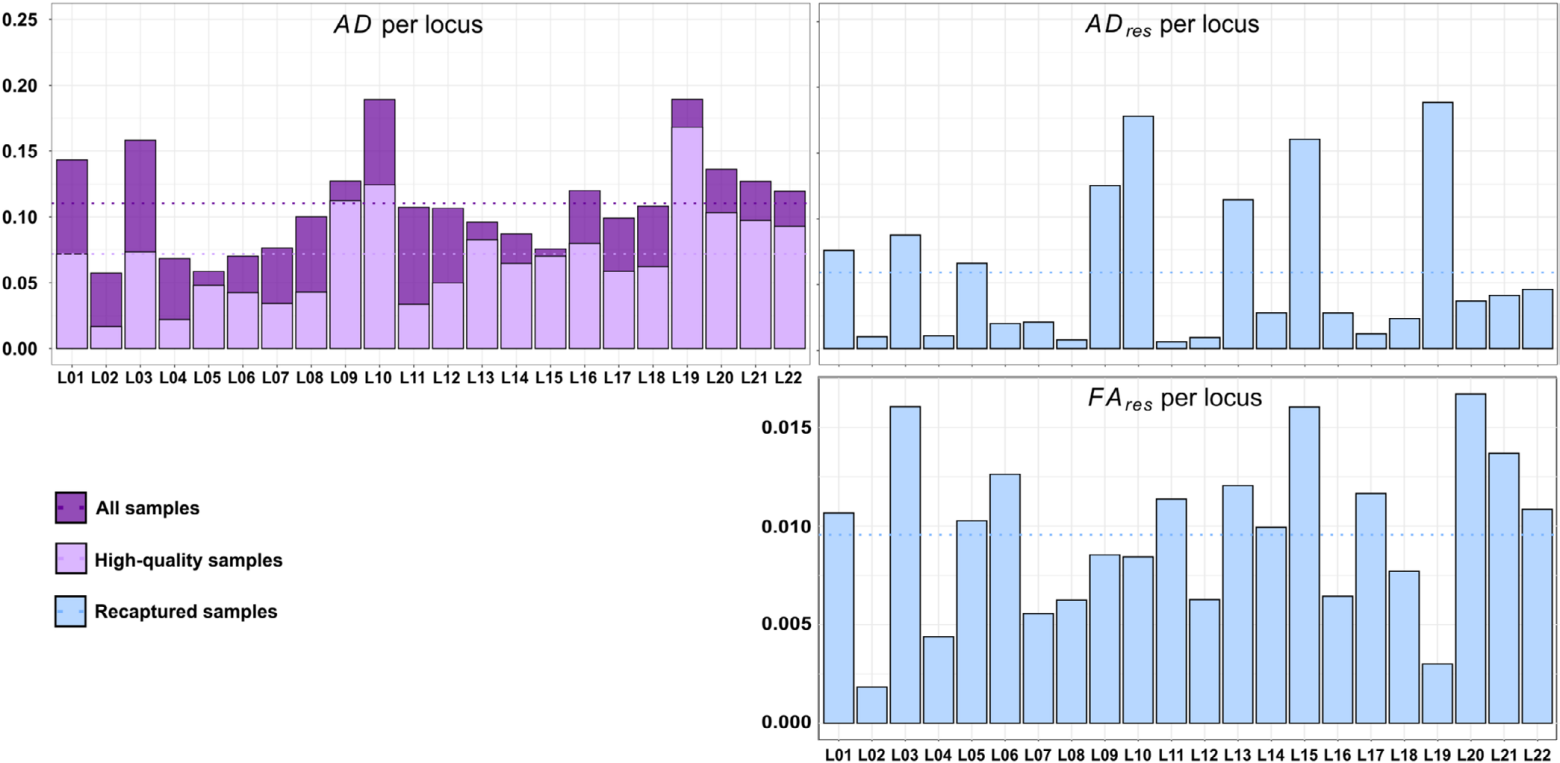
Genotyping error frequencies per locus in wolf samples. For each indicator, darker purple bars indicate values estimated using all genotyped samples (*n* = 5840), while lighter purple bars denote values estimated using only samples with Quality Index (QI) values > 0.5 and without contamination (*n* = 3797) and light blue bars correspond to values estimated on samples from recaptured individuals (*n* = 2743). The mean of the indicator across all loci is represented by the dotted line.

Using the *Sample replicate dataset*, the mean initial AD rate per locus was 11.0%, ranging between 5.73% and 18.92% (Figure 7; Appendix 2). When filtering only replicates from samples with mean QI > 0.5 and no contamination, the mean AD_high_ dropped to 7.1%, with a range per locus between 1.67% and 16.82% (Figure 7).

Using the *Sample consensus dataset* and filtering consensus genotypes of samples from individuals captured several times, AD_res_ dropped down to a mean of 5.8% (range per locus between 0.5% and 18.7%), nearly twofold compared to the mean initial AD per locus (Figure 7; Appendix 2).

FA_res_ was low, varying between 0.2% and 1.7%, with a mean of 1.0% (Figure 7; Appendix 2).

QI values per locus were negatively correlated with the proportion of missing data per locus (Kendall correlation test, *ƭ* = −0.73, *p* = 2.1 × 10^−6^), AD_res_ values (Kendall correlation test, *ƭ* = −0.42, *p* = 0.006), but not with FA_res_ values (Kendall correlation test, *ƭ* = −0.30, *p* = 0.05). Therefore, QI is a good indicator of PCR success and is complementary to genotyping error indicators.

Using the *Individual consensus* dataset and removing the genotype from the individual with a North‐American CR mitotype, Micro‐Checker detected null alleles at Locus 3, Locus 10, and Locus 11. By testing for homozygote excesses and departure from HWE, ML_NullFreq detected null alleles at eight loci (Locus 03, Locus 5, Locus 9, Locus 10, Locus 11, Locus 13, Locus 15, and Locus 22; *p <* 0.001, 10,000 randomizations), with frequencies > 5% (i.e., above the mean AD_res_) in four of these loci (Locus 3, Locus 9, Locus 10, and Locus 11; Appendix 2).

### 4.3 Population Genetic Diversity

Among the 1735 distinct individuals sampled, the sex ratio of 0.55 was significantly biased toward males (*z*‐test, *z‐score* = 16.46, *p* = 4.9 × 10^−5^).

Allelic richness per locus varied between 4 and 16 alleles/locus, with a total of 188 alleles (Table 1). The observed heterozygosity (*H*_*o*_) per locus varied between 0.054 and 0.789, with a mean of 0.482, whereas the expected heterozygosity (*H*_*e*_) per locus varied between 0.056 and 0.796, with a mean of 0.519 (Table 1). We observed significantly high (> 0.1) *F*_IS_ values for five loci (Table 1), indicating a significant departure from HWE. The *F*_IS_ over all loci was 0.072 and slightly differed from 0 (95% confidence interval [CI], 0.065–0.080; *t*‐test, *p* = 0.034). Once the five loci with high *F*_IS_ values (possibly owing to null alleles) were removed, the overall *F*_IS_ decreased to 0.005 and was not significantly different from 0 (95% CI, −0.004 to 0.013). A similar result was obtained when estimating the annual *F*_IS_ values, which were not significantly different from 0 when the five loci with putative null alleles were removed. No year‐to‐year variation was observed in heterozygosity or allelic richness (Appendix 4).

**TABLE 1:** Genetic diversity statistics of the French wolf population using 22 microsatellite loci (*n* = 1735 individuals).

Locus	Name	Na	*H*_*o*_	*H*_*e*_	*F*_IS_
L01	AHT103	7	0.137	0.138	0.019
L02	AHT111	8	0.572	0.578	0.008
L03	AHTk211	8	0.128	0.505	0.758**
L04	FH2096	6	0.557	0.554	−0.013
L05	CPH02	8	0.553	0.583	0.041*
L06	FH2088	7	0.596	0.602	0.017
L07	C09.173	5	0.606	0.626	0.032
L08	CPH05	6	0.646	0.633	−0.02
L09	FH2004	15	0.63	0.724	0.105*
L10	CFX30371	4	0.068	0.13	0.508**
L11	CXX279	8	0.455	0.646	0.306**
L12	C09.250	7	0.734	0.714	−0.031*
L13	FH2161	15	0.673	0.754	0.08*
L14	FH2140	13	0.613	0.619	0.004
L15	INU030	6	0.148	0.151	0.016
L16	FH2137	16	0.779	0.795	0.011
L17	FH2054	9	0.789	0.77	−0.024*
L18	C27.442	6	0.299	0.306	0.025
L19	Dbar1	11	0.051	0.053	0.024
L20	REN162C04	9	0.413	0.4	−0.032
L21	PEZ17	8	0.642	0.653	0.006
L22	FH2010	6	0.446	0.48	0.057*
Total		188	0.479	0.519	0.072*

*Note:* Asterisks indicate loci with significant departure from Hardy–Weinberg equilibrium (*p < 0.05; **p < 0.01).

Abbreviations: *F*_IS_, Inbreeding coefficient; *H*_*e*_, Expected heterozygosity; *H*_*o*_, Observed heterozygosity; Na, Number of alleles.

## 5 Discussion

This study described a standardized and reliable protocol for analyzing noninvasively collected samples for monitoring French wolf populations and providing some basic genetic indices of population diversity.

### 5.1 Methodological Framework to Ensure High‐Quality Genetic Data

Standardized protocols are urgently needed to perform specific analyses and maintain a continuous workflow that is consistent over time, while controlling dropouts and/or false alleles to prevent incorrect genotyping. The protocol described in this study relied on the identification of key steps essential for analyzing noninvasive samples (Creel et al. 2003; Scandura et al. 2006; Stenglein, Waits, et al. 2010; Caniglia et al. 2012; Nakamura et al. 2017; Štikarová et al. 2019). These steps included (i) sampling, which should have excellent coverage of the distribution range of the species under study and high‐quality procedures for sample collection and storage, (ii) selection of the loci used, (iii) successful PCR, (iv) accurate readout of electropherograms, and (v) assessment of remaining genotyping error rates and reliability of genotypes. Once identified, we applied control procedures at each step in a single and continuous workflow to analyze a significant number of samples. To the best of our knowledge, this is the first study using noninvasive samples of gray wolves collected on such a large temporal and spatial scale and applying this range of control procedures.

#### 5.1.1 Sampling

First, the sampling strategy implemented in France stands out as highly intensive monitoring of gray wolf populations on a large scale across an entire country (see, however, the monitoring of the Central European gray wolf population; [Jarausch et al. 2021]). While only a few individuals were observed in 1994, the French wolf population is currently estimated at around 1100 individuals (Duchamp et al. 2023), with a range extending from the French Alps to other mountain ranges in France, such as the Massif Central (Louvrier et al. 2018). The intensity of the sampling strategy has increased with population growth, with approximately 1300–1750 samples per year submitted for genetic analysis since 2017. Therefore, the large number of samples to be analyzed requires a specific and standardized protocol to limit time and financial costs while obtaining a high‐quality dataset (Payne et al. 2018).

Prescreening of samples by sequencing the mtDNA CR serves the dual purpose of identifying nontarget species at the earliest stage and detecting samples with insufficient quantities or degraded DNA, which may hinder successful amplification of the autosomal loci. In this study, we successfully sequenced mtDNA from 82.8% of the collected samples, a success rate consistent with those observed in previous studies and confirming high quality of sample collection, storage, and processing prior to DNA extraction (Appendix 5). Species other than gray wolves (*C. lupus*) were detected in the collected samples, particularly fox (*Vulpes vulpes*, 7.5% of the samples) and domestic dog (*C. familiaris*, 4.9% of the samples), mostly outside the known distribution range. Indeed, the sampling protocol in France advises observers to collect all wolf‐like samples outside the known distribution area to ensure the best possible coverage of dispersed individuals without filtering too many samples and overlooking some detections, thereby compensating for observers' limited experience.

#### 5.1.2 Locus Selection

The number of microsatellite loci used in this study was higher than those used in other studies on wolves (Appendix 5). This improved the discrimination between different individuals, as shown by the low probability of identity among siblings. Although this high number of loci may also increase the proportion of markers with genotyping errors (Mills et al. 2000; McKelvey and Schwartz 2004; Waits and Paetkau 2005), the genotyping error rates estimated here were in a lower range than those of similar studies on wolves, although relatively few microsatellite loci were used (Appendix 5). In addition, relatively few samples were discarded, as shown by the possibility of retaining samples with up to 50% missing loci and still obtaining a PI_sibs_ value < 10^−2^.

While microsatellites have been one of the most commonly used molecular marker types for genetic monitoring programs over the last three decades, new high‐throughput sequencing and genotyping methods have become increasingly affordable, enabling the transition to single nucleotide polymorphism (SNP) loci (Campbell et al. 2015; von Thaden et al. 2017; Eriksson et al. 2020). Unlike microsatellites, these loci do not require calibration between different laboratories, do not rely on electrophoregram reads, and are less impacted by subjective scoring decisions (von Thaden et al. 2017), making them highly valuable for genetic monitoring. Nonetheless, the transition from microsatellite to SNP loci is not always straightforward, especially in long‐term monitoring programs using noninvasive samples, and raises questions about continuity and consistency across time series (Osborne et al. 2023) and sufficient quantity and quality of available DNA (Ogden 2011).

#### 5.1.3 PCR Success

The average PCR success rate was 64.2%, which was in the range of rates observed in other studies with noninvasive samples of gray wolves, and illustrated the high quality of the laboratory procedures (Appendix 5) This also validates the scoring process, even if we were unable to track how many samples were discarded due to disagreements between analysts during electrophoregrams scoring. Relatively high success rates were observed primarily in studies with relatively high‐quality samples, such as those filtering scats < 5‐day‐old, or in studies with a relatively large number of replicates per sample (Appendix 5). The number of replicates varies between studies. The initial recommendation for the multitube approach was to perform eight replicates per sample (Taberlet et al. 1996), which was also based on a low number of loci; however, a relatively high number of replicates were required to ensure the quality of genotyping. Increasing the number of replicates is time‐consuming and costly. Several studies have emphasized the need to weigh the risk between a high number of markers with few replicates, and a low number with a high number of replicates (Frantz et al. 2003; Paetkau 2003; Schwartz et al. 2007). In studies using noninvasive samples of gray wolves, the number of replicates is usually between 2 and 4 per sample (Appendix 5), although in some studies, PCR reactions have been repeated up to 12 times for low‐quality samples (Jarausch et al. 2021). Given the large number of samples analyzed in this study, adjusting the number of replicates after PCR to the quality of each sample (possibly estimated by a prescreening step at 3–4 loci) would be extremely time‐consuming. Additional tests have also shown that samples with low‐quality genotyping in four replicates were usually still low quality with further replicates. Therefore, we decided to perform four replicates for noninvasive samples and two for fresh tissue samples as standards, and discarded all low‐quality samples (QI < 0.5) for further analysis. This strategy enables optimizing costs without investing too much effort in a bad‐quality sample; rather, additional analysis on a new sample from the same area may provide a relatively good‐quality genotype. Notably, direct collection of tissue samples can lead to poor DNA quality if they are collected from animals that have been dead for several days or weeks (89% of tissue samples with QI values < 0.5).

#### 5.1.4 Assessment of Sample Quality and Genotyping Errors

We determined the reliability of the genotypes and selected high‐quality samples by calculating QI, as proposed by Miquel et al. (2006). We differentiated between allelic dropouts and missing data in scoring because allelic dropouts still provide information about the multi‐locus genotype of a sample. Although such indices omit important information about the type and number of errors within a sample or locus (Lampa et al. 2013), they facilitate comparisons between different samples and studies. We decided on a QI threshold of 0.5 to exclude low‐quality samples based on previous analyses and the experience of the genetic laboratory to work on noninvasive samples. This threshold corresponds to the minimum number of loci required to provide PI_sibs_ values < 10^−2^, which comforts this choice and ensures confidence in individual assignments.

We assessed genotyping errors at each stage of analyzing microsatellite loci data: First, for the dataset with all replicates, then for the dataset containing only genotypes sampled multiple times, and finally, for the dataset with one genotype per individual. We did not assess the proportion of false alleles in the replicates at the beginning of the study, which would have been interesting, but they were extremely rare (FAres values between 0.2% and 1.7%). To the best of our knowledge, this is the first study to report all these estimates together, which are usually very confusing when comparing results between different studies. Although we estimated allelic dropout rates from the replicate dataset (mean AD per locus, 11%) that are comparable to other studies on wolves (ranging between 5% and 18%), the residual errors after filtering samples with QI values > 0.5 (mean of 7.1% for AD and of 5.8% for AD_res_) were among the lowest values reported for molecular studies on gray wolves using noninvasive samples (Appendix 5).

While the remaining genotyping error rates were low, we still detected null alleles in the *Individual consensus dataset* (one genotype per individual), with three loci (AHTk211, CFX30371, and CXX279) showing a significant heterozygote deficit and estimated frequencies of null alleles > 10%. One other locus (FH2004) had estimated frequencies > 5%. These heterozygote deficiencies could be owing to null alleles, and these loci could, therefore, be excluded from subsequent analyses of genetic population diversity, as the remaining loci showed sufficient variability. When these loci were removed, the mean and year‐to‐year *F*_IS_ values were no longer significantly different from 0. However, such heterozygote deficiencies could also be owing to population mechanisms, such as social organization or population substructure, which have already been observed in Eurasian wolf populations (Lucchini et al. 2004; Pilot et al. 2006). Further analyses of genetic diversity of populations, considering spatial and temporal structures, should be conducted to further investigate these processes.

### 5.2 Genetic Diversity of the French Wolf Population

The sampling and laboratory procedures conducted over the last 15 years have aimed to describe the main characteristics of the French wolf population. Although further analyses are required to fully describe the population structure of gray wolves in France, some basic indicators and characteristics have been identified.

First, all but six samples, representing 99.9% of the wolf samples, exhibited a single mitotype, w22 (Pilot et al. 2010). The w22 mitotype identified by Pilot et al. (2010) is specific to the Italo–Alpine lineage. Its predominance in the French gray wolf population confirms continuous natural colonization of the French Alps by individuals originating from the Italian Alps and Apennines (Lucchini et al. 2002, 2004; Valière et al. 2003; Fabbri et al. 2007).

The remaining six samples showed the w1 mitotype identified by Pilot et al. (2010), which is specific to the Eastern European lineage. These samples were collected outside the French Alps, mainly during the last 5 years of the study period, and in the northern part of France. These individuals (at least two individuals, but four samples were of too low quality for individual identification) seemed to have migrated from packs in Germany, as confirmed by the German Reference Genetic Laboratory (https://www.senckenberg.de/en/institutes/senckenberg‐research‐institute‐natural‐history‐museum‐frankfurt/division‐river‐ecology‐and‐conservation/cewolf‐consortium/). The increasing frequency of such detections in recent years suggests a new natural route of colonization originating from the Eastern European populations.

Natural colonization of the French Alps was initiated by a few individuals, leading to a moderate bottleneck and loss of genetic diversity (Lucchini et al. 2002; Fabbri et al. 2007). Indeed, the global genetic diversity estimated in the present study was relatively low. The comparison of diversity indices obtained with microsatellite loci in different studies can be difficult because of variations in the loci used (de Groot et al. 2016); but see (Jan et al. 2023). Nevertheless, in this study, we used several previously reported microsatellite loci. Hindrikson et al. (2013) have reported higher observed and expected heterozygosity of gray wolf populations in Estonia and Latvia (between 0.708–0.831 and 0.706–0.807, respectively) at five common microsatellite loci than that in the present study (*H*_*o*_ = 0.599 and *H*_*e*_ = 0.606, respectively). We observed a similar pattern at the six common microsatellite loci used in Poland and Belarus, with an expected heterozygosity of 0.733 (Jędrzejewski et al. 2005) compared with the value of 0.667 in our study. Other studies focusing on the Italian gray wolf population have found diversity indices (*H*_*e*_ between 0.440–0.603 and *H*_*o*_ between 0.490–0.595) (Lucchini et al. 2002; Randi and Lucchini 2002; Fabbri et al. 2007) similar to those reported here. Several studies have also documented lower diversity indices for the Italian gray wolf populations than for other European populations (Lucchini et al. 2004; Randi et al. 2014; Jan et al. 2023), probably because of long genetic isolation of the Italo–Alpine lineage and a recent bottleneck. The dynamics of the French population did not change this trend. Although the French population seem to work well demographically, new migration events from eastern populations have the potential to increase its genetic diversity, as has already been observed in some locations in the Czech Republic (Jędrzejewski et al. 2005; Åkesson et al. 2016; Hulva et al. 2024).

## 6 Conclusions

Noninvasively collected samples are valuable for studying elusive and rare species; therefore, managers are increasingly relying on these data for suitable management plans. Our developed step‐by‐step protocol aims to standardize the analysis of a large number of these samples. It addresses the challenges of sample quality while monitoring noninvasive genetic sampling and improves the cost–benefit ratio of monitoring the French wolf population over a long period of time. By obtaining high‐quality data, this protocol enables subsequent analyses, including the estimation of population size, assessment of hybridization with dogs, and evaluation of population connectivity.

## Author Contributions

**Agathe Pirog:** formal analysis (equal), visualization (lead), writing – original draft (lead), writing – review and editing (lead). **Christophe Duchamp:** conceptualization (equal), funding acquisition (equal), project administration (equal), resources (equal), writing – review and editing (equal). **Cécile Kaerle:** formal analysis (equal), resources (equal), writing – review and editing (equal). **Caroline Dufaure de Citres:** formal analysis (equal), resources (equal), writing – review and editing (equal). **Sabine Rousselot:** formal analysis (equal), resources (equal), writing – review and editing (equal). **Juliette Lavarec:** formal analysis (equal), resources (equal), writing – review and editing (equal). **Guillaume Queney:** conceptualization (equal), formal analysis (equal), funding acquisition (equal), project administration (equal), resources (equal), writing – review and editing (equal).

## Ethics Statement

This study was conducted in compliance with the ethical standards and guidelines for ensuring the welfare of the wolf population in France. This study involved the use of noninvasive samples, such as hair and feces, and tissue samples from wolves found dead or legally shot under Article 16b of the Habitats Directive (Council Directive 92/43/EEC).

## Conflicts of Interest

The authors declare no conflicts of interest.

## Data Availability

The data of the 8733 samples collected in France and consensus genotypes of the 5840 gray wolf samples have been deposited to Dryad, accession number https://datadryad.org/stash/share/uxpxKypphWNA_0MpfHmxWodUeNBHdY1PZgEVD‐1R‐W4.

## Appendix 1 Characteristics of the Microsatellite Loci Used

**Table:**

Locus order	Locus name	Multiplex	Dye	Publication	Type
Sex locus	Amelogenin	Both	VIC/PET	Ji et al. (2007)	Di
1	AHT103	B	6FAM	Godinho et al. (2015)	Di
2	AHT111	B	VIC	Godinho et al. (2015)	Di
3	AHTk211	B	PET	Godinho et al. (2015)	Di
4	FH2096	A	NED	Francisco et al. (1996)	Tetra
5	CPH02	A	6FAM	Godinho et al. (2015)	Di
6	FH2088	B	NED	Francisco et al. (1996)	Di
7	C09.173	A	PET	Godinho et al. (2015)	Di
8	CPH05	A	VIC	Godinho et al. (2015)	Di
9	FH2004	B	6FAM	Francisco et al. (1996)	Tetra
10	CFX30371	B	6FAM	Godinho et al. (2015)	Di
11	CXX279	A	6FAM	Godinho et al. (2015)	Di
12	C09.250	A	VIC	Ostrander et al. (1993)	Di
13	FH2161	A	NED	Godinho et al. (2015)	Tetra
14	FH2140	A	PET	Francisco et al. (1996)	Tetra
15	INU030	B	VIC	Ji et al. (2007)	Di
16	FH2137	A	6FAM	Francisco et al. (1996)	Tetra
17	FH2054	A	NED	Godinho et al. (2015)	Tetra
18	C27.442	B	PET	Godinho et al. (2015)	Di
19	Dbar1	B	NED	Godinho et al. (2015)	Compond
20	REN162C04	B	6FAM	Godinho et al. (2015)	Di
21	PEZ17	A	VIC	Neff et al. (1999)	Tetra
22	FH2010	B	PET	Godinho et al. (2015)	Tetra

## Appendix 2 Quality Index Values and Genotyping Error Frequencies Per Locus in Gray Wolf Samples Collected in France

**Table:**

Locus information		All samples			High quality samples			Recaptured samples		Individual consensus dataset	
Order	Name	PCR success	Mean (QI)	AD	PCR success	Mean (QI)	AD	AD_res_	FA_res_	AD_MC_	AD_ML_
L01	AHT103	75.71%	0.76	14.35%	94.16%	0.94	7.19%	7.45%	1.07%		0.0%
L02	AHT111	77.03%	0.77	5.73%	95.20%	0.95	1.67%	0.90%	0.18%		0.0%
L03	AHTk211	74.04%	0.74	15.82%	91.26%	0.91	7.35%	8.64%	1.60%	x	28.9%***
L04	FH2096	76.65%	0.76	6.85%	96.82%	0.96	2.20%	0.97%	0.44%		1.2%*
L05	CPH02	55.84%	0.57	5.87%	80.24%	0.8	4.80%	6.49%	1.03%		1.9%***
L06	FH2088	67.01%	0.67	7.01%	92.26%	0.92	4.26%	1.90%	1.26%		1.0%
L07	C09.173	70.58%	0.7	7.65%	93.10%	0.93	3.43%	2.03%	0.56%		1.5%*
L08	CPH05	74.47%	0.73	10.02%	95.79%	0.95	4.30%	0.68%	0.63%		0.0%
L09	FH2004	44.74%	0.45	12.71%	65.31%	0.65	11.25%	12.38%	0.85%		5.6%***
L10	CFX30371	69.83%	0.71	18.90%	93.68%	0.94	12.44%	17.65%	0.84%	x	11.9%***
L11	CXX279	75.08%	0.74	10.74%	94.31%	0.94	3.37%	0.52%	1.14%	x	17.6%***
L12	C09.250	72.48%	0.71	10.66%	95.28%	0.94	4.99%	0.84%	0.63%		0.0%
L13	FH2161	35.06%	0.36	9.60%	51.57%	0.53	8.27%	11.31%	1.20%		4.2%***
L14	FH2140	62.26%	0.62	8.71%	87.65%	0.87	6.48%	2.71%	0.99%		0.0%
L15	INU030	60.90%	0.62	7.57%	87.83%	0.88	7.01%	15.90%	1.60%		1.8%***
L16	FH2137	61.93%	0.61	11.99%	86.74%	0.85	7.97%	2.70%	0.64%		0.9%**
L17	FH2054	66.80%	0.66	9.93%	91.34%	0.9	5.88%	1.14%	1.16%		0.0%
L18	C27.442	68.10%	0.68	10.83%	92.77%	0.92	6.22%	2.28%	0.77%		1.0%
L19	Dbar1	59.27%	0.61	18.92%	84.83%	0.85	16.82%	18.71%	0.30%		1.4%
L20	REN162C04	62.99%	0.63	13.61%	88.38%	0.87	10.34%	3.60%	1.67%		0.5%**
L21	PEZ17	57.77%	0.57	12.70%	82.04%	0.81	9.72%	4.04%	1.37%		0.3%
L22	FH2010	56.28%	0.57	11.94%	80.76%	0.8	9.27%	4.50%	1.08%		3.2%***

*Note:* Asterisks Indicate Loci With Significantly Different From Zero Rates of Null Alleles (**p <* 0.05; ***p <* 0.01; ****p <* 0.001).

Abbreviations: AD, Allelic Dropout Rate; AD_MC_, Loci With Null Alleles Detected by Micro‐Checker; AD_ML_, Percentage of Allelic Dropout Estimated by ML_NullFreq; AD_res_, Residual Allelic Dropout Rate; FA_res_, Residual False Allele Rate; QI, Quality Index.

## Appendix 3 Probability of Identity Among Siblings (PI_sibs_) for All Possible Combinations of the 22 Microsatellite Loci

**Table:**

N_Loc_	Min (PI_sibs_)	Q_0.25_ (PI_sibs_)	Mean (PI_sibs_)	Median (PI_sibs_)	Q_0.75_ (PI_sibs_)	Max (PI_sibs_)
2	1.43E‐01	2.24E‐01	3.25E‐01	2.86E‐01	4.06E‐01	8.34E‐01
3	5.68E‐02	1.17E‐01	1.84E‐01	1.64E‐01	2.24E‐01	7.26E‐01
4	2.39E‐02	6.26E‐02	1.04E‐01	8.93E‐02	1.28E‐01	6.20E‐01
5	1.01E‐02	3.36E‐02	5.84E‐02	4.89E‐02	7.29E‐02	4.45E‐01
6	4.72E‐03	1.81E‐02	3.27E‐02	2.70E‐02	4.09E‐02	2.94E‐01
7	2.25E‐03	9.74E‐03	1.82E‐02	1.49E‐02	2.29E‐02	1.75E‐01
8	1.08E‐03	5.29E‐03	1.01E‐02	8.19E‐03	1.28E‐02	9.83E‐02
9	5.27E‐04	2.88E‐03	5.60E‐03	4.51E‐03	7.08E‐03	5.39E‐02
10	2.58E‐04	1.58E‐03	3.09E‐03	2.48E‐03	3.91E‐03	2.81E‐02
11	1.30E‐04	7.81E‐04	1.50E‐03	1.22E‐03	1.90E‐03	1.43E‐02
12	6.64E‐05	4.08E‐04	7.67E‐04	6.30E‐04	9.73E‐04	7.22E‐03
13	3.46E‐05	2.17E‐04	4.02E‐04	3.33E‐04	5.10E‐04	3.54E‐03
14	1.90E‐05	1.26E‐04	2.34E‐04	1.94E‐04	2.98E‐04	1.73E‐03
15	1.07E‐05	7.46E‐05	1.37E‐04	1.15E‐04	1.75E‐04	8.31E‐04
16	6.36E‐06	4.32E‐05	7.58E‐05	6.52E‐05	9.73E‐05	3.95E‐04
17	4.19E‐06	2.49E‐05	4.19E‐05	3.71E‐05	5.36E‐05	1.85E‐04
18	3.01E‐06	1.45E‐05	2.29E‐05	2.08E‐05	2.94E‐05	7.82E‐05
19	2.57E‐06	8.36E‐06	1.24E‐05	1.13E‐05	1.60E‐05	3.29E‐05
20	2.24E‐06	4.59E‐06	6.65E‐06	6.57E‐06	8.40E‐06	1.31E‐05
21	1.98E‐06	2.83E‐06	3.53E‐06	3.70E‐06	4.00E‐06	5.05E‐06
22	1.87E‐06	1.87E‐06	1.87E‐06	1.87E‐06	1.87E‐06	1.87E‐06

Abbreviation: *N*_Loc_, Number of Loci Included in the Combination.

## Appendix 4 Annual Genetic Diversity Statistics of the French Wolf Population Using 17 Microsatellite Loci (*N* = 1735 Individuals)

**Table:**

Year	*N*	Na	Ar	*H*_*e*_	*H*_*o*_	*F*_IS_
2006	8	2.41	2.35 ± 0.84	0.406 ± 0.228	0.419 ± 0.288	0.04 (−0.26 to 0.15)
2007	16	2.76	2.41 ± 0.92	0.401 ± 0.240	0.459 ± 0.283	−0.11 (−0.25 to −0.06)
2008	18	2.71	2.36 ± 0.87	0.422 ± 0.234	0.421 ± 0.238	0.03 (−0.13 to 0.11)
2009	17	2.82	2.40 ± 0.85	0.425 ± 0.219	0.480 ± 0.277	−0.10 (−0.24 to −0.02)
2010	16	2.76	2.35 ± 0.83	0.405 ± 0.222	0.432 ± 0.259	−0.03 (−0.18 to 0.03)
2011	29	3.94	2.78 ± 1.02	0.473 ± 0.233	0.494 ± 0.260	−0.03 (−0.10 to 0.01)
2012	27	3.65	2.73 ± 0.95	0.489 ± 0.224	0.493 ± 0.241	0.01 (−0.09 to 0.07)
2013	33	3.71	2.62 ± 0.91	0.469 ± 0.231	0.472 ± 0.258	0.01 (−0.08 to 0.06)
2014	80	4.65	2.72 ± 0.98	0.475 ± 0.236	0.472 ± 0.243	0.01 (−0.04 to 0.05)
2015	119	5.00	2.82 ± 0.95	0.494 ± 0.220	0.501 ± 0.224	−0.01 (−0.05 to 0.02)
2016	162	5.47	2.74 ± 0.99	0.478 ± 0.236	0.471 ± 0.243	0.02 (−0.01 to 0.04)
2017	227	5.65	2.87 ± 1.00	0.502 ± 0.229	0.508 ± 0.240	−0.01 (−0.03 to 0.02)
2018	284	5.71	2.87 ± 0.94	0.504 ± 0.220	0.502 ± 0.220	0.01 (−0.02 to 0.03)
2019	353	6.06	2.87 ± 0.97	0.504 ± 0.225	0.504 ± 0.230	0.00 (−0.02 to 0.02)
2020	383	6.71	2.94 ± 1.01	0.520 ± 0.224	0.513 ± 0.224	0.01 (−0.01 to 0.03)
2021	437	6.65	2.92 ± 0.98	0.521 ± 0.224	0.523 ± 0.228	0.00 (−0.02 to 0.01)
2022	143	5.24	2.92 ± 0.98	0.517 ± 0.230	0.523 ± 0.242	−0.01 (−0.04 to 0.02)

*Note:* The Five Microsatellite Loci With Putative Null Alleles Were Removed Prior to Analysis and Genotypes of Recaptured Individuals Were Included Once per Year. The mean ± standard deviation across the 17 loci is given for each index while the 95% confidence interval is indicated in brackets for the inbreeding coefficient.

Abbreviations: Ar, Rarefied Allelic Richness (20 Alleles); *F*_IS_, Inbreeding Coefficient; *H*_*e*_, Expected Heterozygosity; *H*_*o*_, Observed Heterozygosity; *N*, Number of Individuals; Na, Number of Alleles.

## Appendix 5 Genotyping Errors Estimated in Studies Using Noninvasively Collected Samples and Focusing on the Gray Wolf

**Table:**

Study	*n*	Nmsat	Prescreening (PS)	PS+	Nrep	PCR+	Samples selection (SS)	SS+	AD	FA
This study	**8733**	**22**	**mtDNA**	**82.8**	**2–4**	**64.1**	**QI > 0.5 and no contamination**	**65**	**7.1**	—
Caniglia et al. (2012)	4839	12	Two loci	62	4		At least 50% amplification prescreening step and *R* > 0.95	45.0	**14.0**	**0.9**
Caniglia, Fabbri, Greco, et al. (2013)	356	13	Two loci		4–8		At least 50% amplification prescreening step and *R* > 0.95	49.0	**15.2**	**3.10**
Caniglia, Fabbri, Mastrogiuseppe, et al. (2013)	33	12	Four replicates at all loci	42	4–8	58	At least 50% amplification prescreening step and *R* > 0.95	55.0	**19.0**	**2.6**
Canu et al. (2017)	24	11			3	76.9	At least 30% amplification	79.0	4.1	
Creel et al. (2003)	288	13					No Contamination Or Poor Amplification	78.8	**11.1**	**5.6**
Dufresnes et al. (2019)	3468	11	mtDNA	89.6	8			53.1	5.8	0.9
Fabbri et al. (2012)	46	6	Two loci		4–8	90	At least 50% Amplification Prescreening Step	46.0	**19.0**	**2.0**
Galaverni et al. (2012)	103	12	Four replicates at three loci		4–8		At least 50% Amplification Prescreening Step And *R* > 0.95	48.5	**20.0**	**1.0**
Godinho et al. (2015)	38	13	mtDNA +4 replicates at three loci	92.1	4		100% Amplification At Prescreening Step		**12.3**	**1.0**
Hausknecht et al. (2007)	41	6	qPCR	80	3	58			2.1	4.2
Hausknecht et al. (2010)	39	8			3	93	At least eight Loci With Amplification And No Suspicious AD Or FA	51.3	**13.4**	**3.7**
Jarausch et al. (2023)	444	13	mtDNA		4–8			66.7	5.7	0.8
Lucchini et al. (2002)	130	9	mtDNA	84	2–6	69	100% amplification after six replicates	53.0	**18.3**	**0.3**
Nakamura et al. (2017)	713	13	mtDNA +4 replicates at four loci	79.5	4	29.7	100% amplification at prescreening step	41.7	**11.2**	**0.7**
Nakamura et al. (2021)	1000	19	mtDNA +4 replicates at four loci	81	4		100% amplification at prescreening step	47.6	**10.6**	**0.7**
Pacheco et al. (2017)	332	17	mtDNA +4 replicates at four loci	91	4		At least 60% Amplification Prescreening Step And 80% Amplification After	61.0	**3.3**	**0.5**
Salvatori et al. (2019)	195	16	mtDNA +3 loci	48	4		At least 75% Amplification		**9.5**	**0.2**
Santini et al. (2007)	11	6			4	78.3			**12.0**	**2.0**
Scandura (2005)	37	10	Four loci		3	77.2	100% Amplification	70.0	5.0	0.9
Scandura et al. (2006)	269	10			**				**2.9**	**1.6**
Stansbury et al. (2014)	2103	9	mtDNA +2 replicates at all loci	92	2–5		At least five Loci With Amplification Prescreening Step	66.0	**8.0** ^**a**^	**0.8** ^**a**^
Stenglein, Waits, et al. (2010)	1585	8–9	mtDNA +2 replicates at all loci	89	3–5	76.5	At least five Loci With Amplification Prescreening Step	51.5	**16.5**	**4.0**
Stenglein, De Barba, et al. (2010)	30	8	Two replicates at all loci		> 2	66	At least 50% Amplification Prescreening Step	55.5	**19.0**	**3.0**
Štikarová et al. (2019)	39	*			4				35.4	
Szewczyk et al. (2019)	2347	13	Two replicates at all loci		2–4		At least 65% Amplification Prescreening Step And nine loci With Amplification After	64.5	3.6	

*Note:* In bold are AD and FA values estimated using the same methodology as this study. *Panels A and B MiniDogFiler. **Until consensus is reached. ^a^Two replicates of 100 randomly selected samples used to estimate genotyping error rates.

Abbreviations: AD, Allelic Dropout Rate (In %); FA, False Allele Rate (In %); *N*, number of samples; Nmsat, number of microsatellite loci; Nrep, number of replicates per sample; PCR+, PCR Success (In %); PS+, Success Prescreening Step (In %); SS+, Samples With Successful Genotyping After Selection Step (In %).
